# Determination of volatile marker compounds of common coffee roast defects

**DOI:** 10.1016/j.foodchem.2016.04.124

**Published:** 2016-11-15

**Authors:** Ni Yang, Chujiao Liu, Xingkun Liu, Tina Kreuzfeldt Degn, Morten Munchow, Ian Fisk

**Affiliations:** a Division of Food Sciences, University of Nottingham, Sutton Bonington Campus, Loughborough LE12 5RD, United Kingdom; b Department of Food Science, Faculty of Science, University of Copenhagen, Rolighedsvej 30, 1958 Frederiksberg C, Denmark; c The Specialty Coffee Association of Europe, Chelmsford, Essex, United Kingdom; d CoffeeMind Aps, Hansstedvej 35, 2500 Valby, Denmark

**Keywords:** Coffee, Roast defects, Coffee production, Aroma chemistry, Quality control

## Abstract

Coffee beans from the same origin were roasted using six time-temperature profiles, in order to identify volatile aroma compounds associated with five common roast coffee defects (light, scorched, dark, baked and underdeveloped). Thirty-seven volatile aroma compounds were selected on the basis that they had previously been identified as potent odorants of coffee and were also identified in all coffee brew preparations; the relative abundance of these aroma compounds was then evaluated using gas chromatography mass spectrometry (GC–MS) with headspace solid phase micro extraction. Some of the 37 key aroma compounds were significantly changed in each coffee roast defect and changes in one marker compound was chosen for each defect type, that is, indole for light defect, 4-ethyl-2-methoxyphenol for scorched defect, phenol for dark defect, maltol for baked defect and 2,5-dimethylfuran for underdeveloped defect. The association of specific changes in aroma profiles for different roast defects has not been shown previously and could be incorporated into screening tools to enable the coffee industry quickly identify if roast defects occur during production.

## Highlights

• Coffee was roasted and five typical roasted defects were replicated.
• Light roast defect had increased indole.
• Scorched roast defect had increased 4-ethyl-2-methoxyphenol.
• Dark and baked roast defect had increased phenol and maltol respectively.
• Underdeveloped roast defect had increased 2,5-dimethylfuran.

## 1 Introduction

Coffee is one of the most popular hot beverages consumed around the world. It is drunk by millions of people every day and there continues to be an increasing demanding for high quality speciality coffees (Bhumiratana, Adhikari, & Chambers Iv, 2011). The production of coffee that is perceived to be of good quality is dependent on many factors, these include the quality of the green bean, roaster type, the extraction process and water type used during brewing (Ribeiro, Augusto, Salva, Thomaziello, & Ferreira, 2009). Furthermore, coffee’s unique aroma profile is very closely related to the time-temperature profiles used during the roasting process (Baggenstoss et al., 2008, Fisk et al., 2012, Gloess et al., 2014).

Many different methods have been proposed to determine the optimum degree of roast. These include colour generation, weight loss, moisture content, degradation of chlorogenic acid or the ratio of free amino acids (Baggenstoss et al., 2008). However, the nature of the roasting process is very complex and no clear universally accepted definitions exist. Colour, although imprecise, is therefore currently used as the industry standard (Şenyuva & Gökmen, 2005).

Unlike flavour defects which result directly from the green bean, its production, processing and storage (Mancha Agresti, Franca, Oliveira, & Augusti, 2008), the term roast defects indicates problems within the roasting process, directly resulting in the presence of off-flavours in the coffee brew. Variations in time-temperature profiles within the roasting process will directly impact the rate of moisture loss, internal bean temperature and local microchemistry. This will regulate the rate at which caramelisation, Maillard chemistry, oxidation and pyrolysis occurs, and the resultant development of colour and flavour in the final roasted coffee bean (Sunarharum, Williams, & Smyth, 2014).

More than 800 volatile compounds have been identified to be present in roast and ground coffee. The most common classes of compounds reported in the headspace include acids, aldehydes (Ullrich & Grosch, 1987), alcohols (Merritt & Robertson, 1966), sulphur compounds (Silwar, 1986), phenolic compounds (Pypker & Brouwer, 1970), pyrazines (Reymond, Muggler-Chavan, Viani, Vuataz, & Egli, 1966), pyridines (Balts & Bochmann, 1987), thiophenes (Vitzthum & Werkhoff, 1976), pyrroles and furans. Due to the high number of compounds and inherent complexity of aroma chemistry within the coffee bean, it is essential to have both a methodology and a source of markers available to the coffee industry to enable them to identify roast defects.

The overarching objective of this study was to demonstrate how aroma profiles were impacted when roast defects occurred during coffee roasting and to generate a list of marker compounds associated with five roast defects (light, scorched, dark, baked and underdeveloped). Gas chromatography mass spectrometry (GC–MS) with headspace solid phase micro extraction (SPME) was used to compare the changes in volatile aroma compounds present in the roast defect coffee. To the best of our knowledge this is the first study to present a methodology for the evaluation of, and generate a framework of compounds associated with, roast defects in the aroma profile of roasted coffee.

## 2 Materials and methods

### 2.1 Coffee samples

All the coffee beans were single-origin washed Kenya Arabica from the wet mill from crop years 2012/2013 and 2013/2014. They were supplied by Kontra Coffee (Dag Hammarskjölds Alle 36, 2100 Copenhagen, Denmark), and were roasted using a batch size of 1 kg through a Probat drum roaster (Probat-Werke, Germany) modified to include additional temperature probes to monitor bean temperature. Roast degree was measured with a Javalytics JAV-RDA-DN (Madison Instruments, Inc., United States) and Agtron number was used to indicate the colour of the roast- the smaller the number, the darker the roast.

The roasting parameters for the standard roast and five roasting defects were recorded (Table S1). When a popping sound is perceived during roasting, it is the first crack and the development time is defined as the time from first crack to the end of roasting in this study. The standard roasting started when the air temperature in the roaster was at 210 °C and its developing time was 2 min 40 s with the total roasting time of 11 min 25 s. The light roast defect had the same starting temperature (210 °C) but with only 10 s development time and total roasting time of 8 min 40 s. The scorched roast defect had a higher starting temperature (275 °C) and shorter total roasting time (7 min 40 s) than standard roast. The dark roast defect had longer developing time (4 min 45 s) than the standard roast, and resulted in an additional 2 min of total roasting time. Baked roast defect had a higher initial temperature (230 °C) than the standard roast, and longer development time (6 min 20 s). In the underdeveloped samples, coffee was roasted at a much lower initial temperature (135 °C) and a longer total roasting time (20 min 20 s) than the standard roast.

Roasted samples were individually packed in the odour-free air-tight package. Beans from each type were weighed (90 g) and ground using an electronic coffee grinder (KG 49, Delonghi, Australia), then passed through a metal sieve (710 mm, Endecotts, Essex, UK). Ground coffee (11 g) was brewed with boiling water (200 mL) using a French press brewer (3 Cup Black Cafetiere, Argos, UK) using deionised water (Purite Ltd, Oxon, UK). The resulting coffee brew was stirred 5 times with a spoon and allowed to stand for 4 min before depressing the plunger. The brewed coffee (4 mL) was stored in amber glass vials (20 mL, 22.5 mm × 75.5 mm, Sigma-Aldrich, UK) and closed with crimp seals (Sigma-Aldrich, UK) for GC–MS analysis. Each sample type had four replicates and they were analysed in a randomised order by GC–MS.

### 2.2 Gas chromatography–mass spectrometry

A trace 1300 series Gas Chromatograph coupled with the Single-Quadrupole Mass Spectrometer (Thermo Fisher Scientific, Hemel Hemptead, UK) was used for analysis of volatile aroma compounds. Samples were incubated at 55 °C for 5 min with shaking. A 50/30 μm DVB/CAR/PDMS SPME Fibre (Supelco, Sigma Aldrich, UK) was used to extract volatile aroma compounds from the sample headspace (extraction for 5 min then desorption for 15 min). The inlet temperature was set at 200 °C and a splitless mode was used, and the constant carrier pressure was at 103 kPa.

Separation was carried out on a ZB-WAX Capillary GC Column (length 30 m, inner diameter 0.25 mm, and film thickness 1 μm; Phenomenex Inc., Macclesfield, UK). Column temperature was held initially at 40 °C for 5 min, increased by 3 °C/min to 180 °C, then 8 °C/min to 240 °C and held for 2 min. Full scan mode was used to detect the volatile compounds (mass range from 20 to 300 AMU).

Volatiles were identified by comparison of each mass spectrum with either the spectra from authentic compounds or with spectra in reference libraries (NIST/EPA/NIH Mass Spectral Library, version 2.0, Faircom Corporation, U.S.). The relative abundance of each volatile compound present in the headspace was calculated by its GC peak area found in the defect roast (average of 4 replicates), normalised to its respective peak found in the standard roast as 100%, and presented as a percentage. All samples were analysed in one run in randomised order with external standards run prior to and after the run.

### 2.3 Statistical analysis

Relative abundance of each aroma compound was analysed by ANOVA to identify if a significant difference (p < 0.05) present for each compound between each defect roast and standard roast (IBM® SPSS® Statistics version 21.0.0). Principle Component Analysis (PCA) was generated using the Unscrambler® X version 9.1.2 (Camo Software, Oslo, Norway).

## 3 Results and discussion

### 3.1 Aroma compounds of roasted coffee samples

Coffee samples were generated with roasting parameters to represent five different types of roast defects. Thirty-seven aroma compounds were identified within the coffee samples and these compounds were selected as initial target compounds for the determination of the roast and ground coffee aroma profiles (Table 1). All of the compounds have previously been identified in coffee. The range of compounds were classified into 10 groups based on the chemical properties of the compound (4 organic acids, 2 alcohols, 2 ethers, 4 aldehydes, 5 ketones, 2 phenolic compounds, 3 furans, 4 sulphide compounds, 7 pyrazines, and 4 N-containing heterocyclic compounds). Literature descriptions of their aroma profile varied from malty, nutty, grassy, sour, burnt, and smoky.

### 3.2 Aroma chemistry of roast coffee defects

#### 3.2.1 Light roast defect

**Fig. 1:**
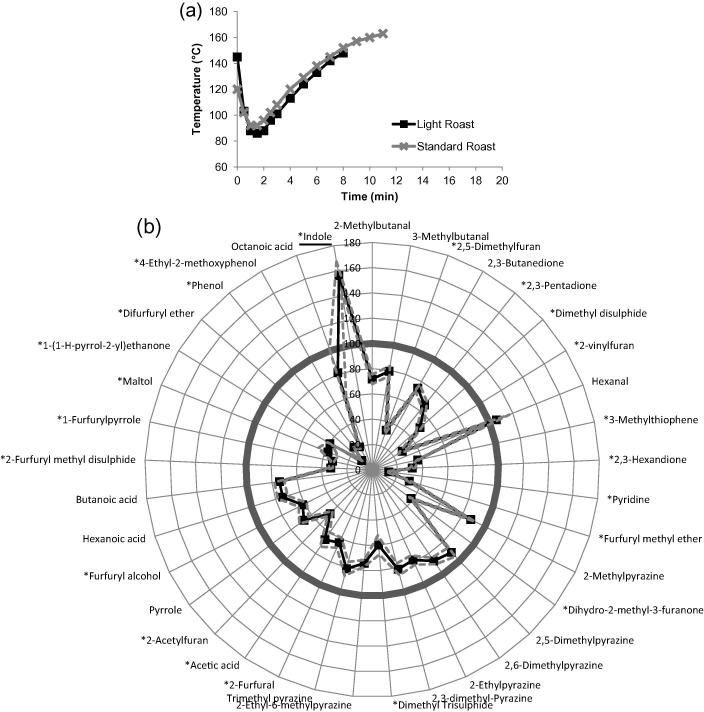
Light roast defect compared with standard roast: a) bean temperature during roasting for standard roast (grey line with cross markers) and light roast defect (black line with square markers); b) aroma profile between standard roast (smooth circle at 100%) and light roast defect (spider diagram with square markers). Compounds with ∗ had a significant difference (p < 0.05). Marker compound was underlined.

The bean temperature was evaluated for both a standard roast and a light roast defect, this is shown in Fig. 1 a. Light roast defect samples were subjected to a similar temperature profile as the standard roast, but the bean had higher bean temperature at 160 °C than standard roast (120 °C) and the roasting process was stopped around 4 min earlier. During roasting, green beans undergo several stages including the endothermic heating up take phase, followed by the larger exothermic heat release phase. The key stage is from the first crack, when a popping sound is perceived, to the second crack (Gloess et al., 2014). So the development time is essential for aroma generation during roasting.

The impact of shortening the development time on the resulting aroma profile was determined and is presented in Fig. 1b. In general, there was a reduction in most volatile aroma compounds in the light roast defect, suggesting that the shorter development time did not allow full aroma development to occur and that the aroma generation reaction was terminated prematurely.

There was a significant elevation in the relative abundance of indole in the light roast defect coffee (p < 0.05). Indole has been previously identified in both green coffee (Cantergiani et al., 2001) and roasted coffee (Dorfner, Ferge, Yeretzian, Kettrup, & Zimmermann, 2004) and is proposed to originate from the waxes surrounding the coffee beans (Arctander, 1969). Due to elevated abundance in the light roast defect samples, indole is therefore proposed as a chemical marker for the light roast defect.

In addition, there was a significant reduction in the abundance of difurfuryl ether (p < 0.05) and pyridine (p < 0.05). Difurfuryl ether is known to increase as roasting time increases (López-Galilea, Fournier, Cid, & Guichard, 2006); whereas, pyridine is also formed during longer roasting conditions and has previously been proposed to originate during roasting through the decomposition of trigonelline (Zheng & Ashihara, 2004) and by Maillard chemistry (Mottram, 2007). Low levels of difurfuryl ether and pyridine, and high levels of indole within roasted coffee samples may therefore indicate reduced thermal degradation and a shorter development time.

#### 3.2.2 Scorched roast defect

**Fig. 2:**
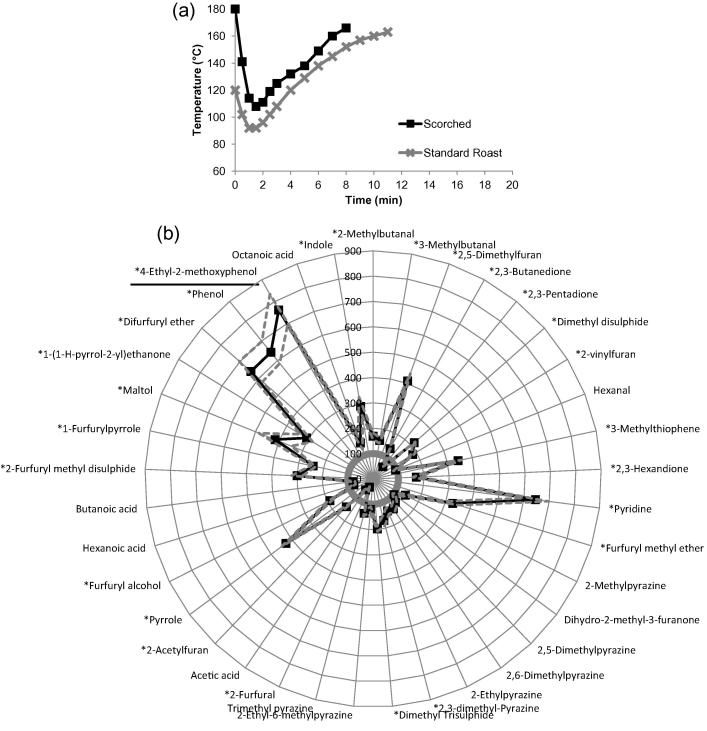
Scorched roast defect compared with standard roast: a) bean temperature during roasting for standard roast (grey line with cross markers) and scorched roast defect (black line with square markers); b) aroma profile between standard roast (smooth circle at 100%) and scorched roast defect (spider diagram with square markers). Compounds with ∗ had a significant difference (p < 0.05). Marker compound was underlined.

This defect resembled the standard roast profile, but was a quicker process and occurred at a higher temperature (Fig. 2a). There was a major change in the aroma profile of the scorched roast coffee when compared to the standard roast coffee (Fig. 2b) due to the high temperature-short time roasting profile.

When comparing the relative abundance of the compounds, 4-ethyl-2-methoxyphenol was shown to increase significantly (p < 0.05) due to the scorched roast defect. This compound, which can be formed as a consequence of the thermal decomposition of ferulic acid (Fiddler, Parker, Wasserman, & Doerr, 1967), is therefore proposed as a marker for the scorched roast defect. Three other compounds (pyridine, phenol and difurfuryl ether) increased in abundance 6-fold when compared to the standard roast (p < 0.05).

Conversely, a significant reduction in 2-furfural was observed in the scorched roast defect when compared to the standard roast (p < 0.05). Other researchers (Silwar & Lüllmann, 1993) have identified that 2-furfural was formed after 5 min at 230 °C, but decomposed at a high temperature, which is consistent with our findings.

#### 3.2.3 Dark roast defect

**Fig. 3:**
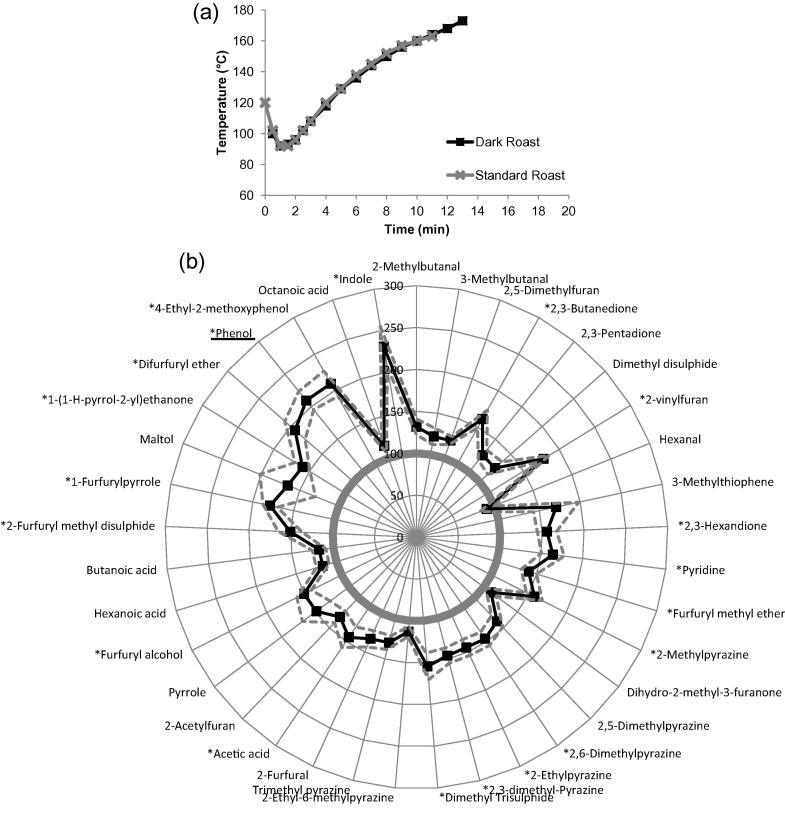
Dark roast defect compared with standard roast: a) bean temperature during roasting for standard roast (grey line with cross markers) and dark roast defect (black line with square markers); b) aroma profile between standard roast (smooth circle at 100%) and dark roast defect (spider diagram with square markers). Compounds with ∗ indicated a significant difference between scorched roast and standard roast (p < 0.05). Marker compound was underlined.

This defect had a similar roasting profile with an extended 2 min roasting time when compared to the standard roast (Fig. 3a). The relative abundance of the resulting volatile aroma compounds (Fig. 3b) was generally increased when compared with the standard roast but without the significant distortion observed in scorched roast defect samples.

The most significant compounds that were modified during the dark roast defect were indole, 4-ethyl-2-methoxyphenol and phenol when compared with a standard roast (p < 0.05). A longer roasting profile also resulted in a significant increase in most pyrazines, which are directly produced via the Maillard reaction between reducing sugars and amino acids during coffee roasting (Pittet & Hruza, 1974). This study confirmed the positive correlation between roasting time and levels of pyrazine generated.

Analysis revealed that most concentrations of compounds increased in the dark roast defect, apart from hexanal which was present at 90% of the level found in the standard roast profile (Fig. 3b). Although the reduction was not significant in our experiment, Ramos, Valero, Ibáñez, Reglero, and Tabera (1998) found that hexanal decreased after roasting, and it is possible that use of higher roasting temperatures and longer times may have induced a more extensive degradation of aroma compounds or that it may be lost due to volatility.

#### 3.2.4 Baked roast defect

**Fig. 4:**
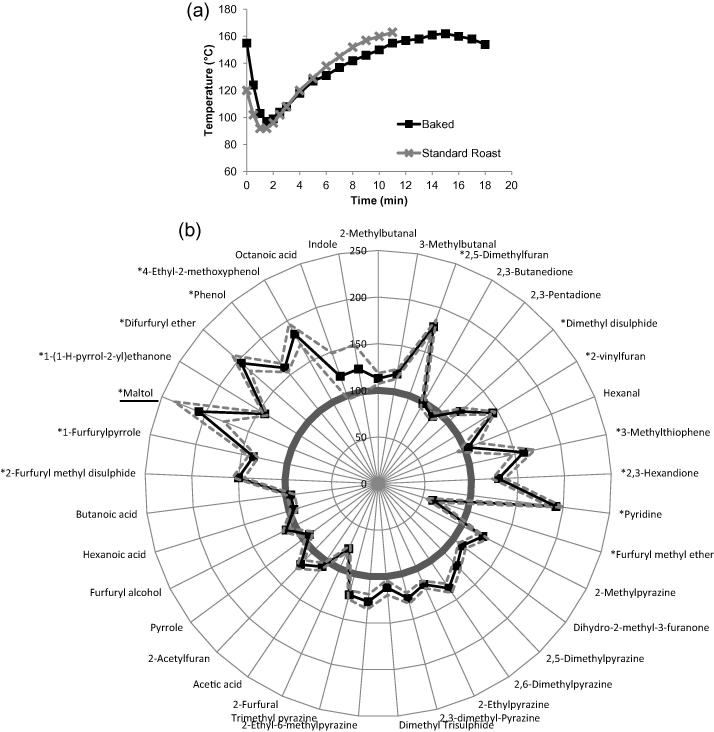
Baked roast defect compared with standard roast: a) bean temperature during roasting for standard roast (grey line with cross markers) and baked roast defect(black line with square markers); b) aroma profile between standard roast (smooth circle at 100%) and baked roast defect (spider diagram with square markers). Compounds with ∗ indicated a significant difference between scorched roast and standard roast (p < 0.05). Marker compound was underlined.

Baked roast defect had a higher initial temperature in the bean (160 °C) than the standard roast bean (120 °C), and its roasting time was extended for about 6 min extra (Fig. 4a). The resulting aroma profile revealed a slight increase in most compounds measured (Fig. 4b). The top 3 compounds with the largest increase were maltol, difurfuryl ether, and pyridine when compared to the standard roast (p < 0.05). Maltol, was selected as the marker for this roast defect, and it can be formed from the degradation of maltose and Amadori intermediates (Flament, 2002), was also previously reported to be present at increased concentration in over-roasted coffee by Silwar and Lüllmann (1993).

Additionally, the identified furan derivatives (2-vinylfuran and 2,-5 dimethylfuran) were shown to be present at a higher concentration in the baked defect when compared to the standard roast profile (p > 0.05). Furans can be formed from the thermal degradation of d-glucose and sugar polymers (Heyns, Stute, & Paulsen, 1966) and have also been shown to be formed during the thermal oxidation of lipids, from thiamine degradation and from the breakdown of nucleotides (Mottram, 2007).

Conversely, furfuryl methyl had a significant lower concentration in the baked roast defect when compared with standard roast (p < 0.05). Furfuryl methyl ether is also known as 2-(methoxymethyl) furan and, on its own, is proposed to have a strong nutty and coffee grounds-like taste (Arctander, 1969).

#### 3.2.5 Underdeveloped roast defect

**Fig. 5:**
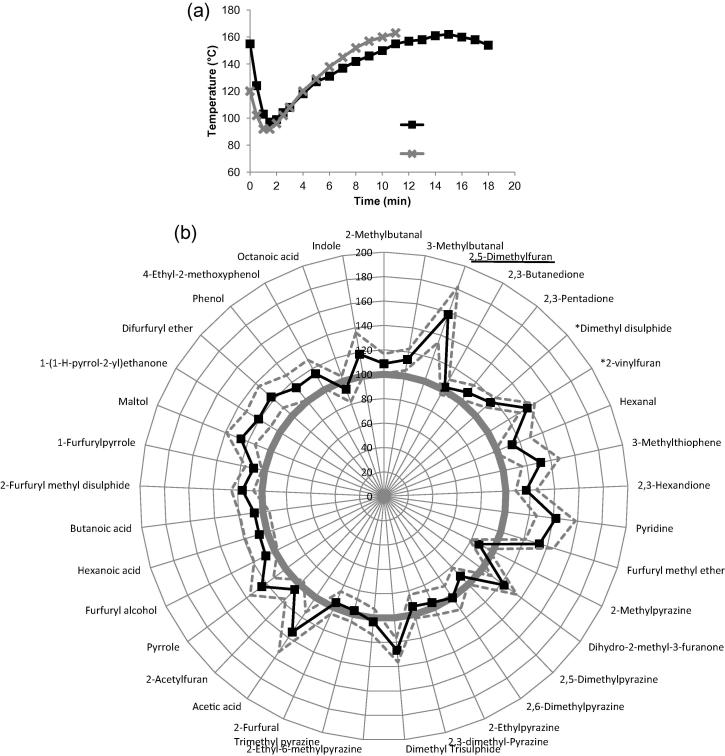
Underdevelopment roast defect compared with standard roast: a) bean temperature during roasting for standard roast (grey line with cross markers) and underdevelopment roast defect (black line with square markers); b) aroma profile between standard roast (smooth circle at 100%) and underdevelopment roast defect (average value showed in black solid with square markers, with +/− standard errors showed in grey dotted lines). Compounds with ∗ indicated a significant difference between scorched roast and standard roast (p < 0.05). Marker compound was underlined.

In the underdeveloped samples, coffee was roasted at a slower rate with a much lower initial temperature with bean temperature at 80 °C and more than 8 min longer roast time (Fig. 5a). In the underdeveloped samples (Fig. 5b), the aroma compound with the largest increase was 2,5-dimethylfuran when compared with a standard roast (p < 0.05), and it could be the marker for this roast defect. 2,5-dimethylfuran is regarded as being important for dark-roasted coffee, as noted previously (Leino, Kaitaranta, & Kallio, 1992) and it is formed by the thermal degradation of glucose (Heyns et al., 1966). On its own, 2,5-dimethylfuran is known to have an ethereal or solvent-like pungent odour (Arctander, 1969), which may contribute to the characteristic flavour of these defect samples.

There was a slight decrease in presence of some of the pyrazines (trimethyl pyrazine, 2-ethylpyrazine, 2,3-dimethyl-pyrazine, and 2-methyl pyrazine), but not significant (p > 0.05) . It is interesting to note that despite the low initial roasting temperature (135 °C compared to standard roast 210 °C), the relative abundance of most measured aroma compounds is roughly comparable.

### 3.3 Summary of all coffee samples

To compare overall aroma profiles across all the coffee samples, Principle component analysis (PCA) was applied to all coffee samples for all 37 volatile aroma compounds (Fig. S2). The first principle component (PC1) accounted for 71% of the variance and the second principle component (PC2) accounted for 20% of the variance in the data, indicating the PCA offered discrimination and effective illustration of the variation among the samples. The greatest difference was observed between the light roast defect and scorched roast defect (PC1). In general, there was an increase in aroma compound concentration in the dark and scorched brews and a reduction in the light roast defect. Underdeveloped, baked and standard roast defects were centrally located in the centre of the bi-plot and showed no clear differentiation across the principle components presented. The second principle component (PC2) explained 20% of the variance, and resolved the dark roast defect from the light and scorched roast defects, a dark defect positively correlated with the three acids acetic acid, butanoic acid and hexanoic acid, which conversely were negatively correlated with the scorched and light roast defect process. Both light and scorched samples had very short development time (less than 2 min), which confirmed that the development time from first crack to finish is a critical part of the time-temperature profile during coffee roasting.

Whilst aroma chemistry development during coffee roasting is extremely complex, marker compounds were selected that correlated with each roast coffee defect. These are summarised in Table 1 and are presented such that future workers could build on the data presented to explain differences in future work, or to target the development of roasting profiles.

In general, low roast intensity are associated with higher levels of organic acids (acetic acid, butanoic acid, hexanoic acid), and with greater roast intensity these acids are lost, whilst Maillard chemistry and lipid breakdown products are formed to a great extent.

However, different species, varieties and geographical origin of coffee samples need to be evaluated to confirm the ultimate selection of these marker compounds and this work should be replicated for different roasting types. Furthermore, the work does not include sensory characterisation, as due to the multimodal effect of complex aroma-taste profiles we do not anticipate that these compounds are the sole drivers of perceptual differences within the roast defects, but we believe that they could be used to target analytical strategies to characterise roast defects and develop engineering solutions to standardise roast conditions and mechanistically explain the underlying drivers controlling Maillard Chemistry, lipid oxidation, caramelisation and pyrolysis occurring during roasting.

## 4 Conclusion

The study illustrated how aroma profiles were impacted when five common roast defects occurred during coffee roasting. The five specific roast defects (light, scorched, dark, baked and underdeveloped) were shown to have elevated levels of indole, 4-ethyl-2-methoxyphenol, phenol, maltol and 2,5-dimethylfuran respectively. The association of specific changes in aroma profiles for different roast defects has not been shown previously and could be incorporated into screening tools to enable the coffee industry quickly identify if roast defects occur during production, although it should be noted that the specific marker compounds may change with variations in green bean chemistry due to species and production techniques. Sensory studies have been carried out to confirm if those chemical changes are perceivable, and the results will be published in a future publication.

## Figures and Tables

**Table 1:** Detection of 37 volatile aroma compounds in the standard roast coffee and defect samples.

	Retention time	Aroma compound	Odour description^a^	Functional group
1	2.25	3-Methylbutanal	Malty	Aldehyde
2	2.28	2-Methylbutanal	Malty	Aldehyde
3	2.70	2,5-Dimethylfuran	Ethereal	Furan
4	3.08	2,3-Butanedione	Buttery, cheesy	Ketone
5	4.88	2,3-Pentadione	Oily buttery	Ketone
6	5.00	Dimethyl disulphide	Onion	Sulphide
7	5.22	2-vinylfuran	Ethereal, rum, cocoa note	Furan
8	5.35	Hexanal	Grassy, green oily	Aldehyde
9	5.54	3-Methylthiophene	Ash	Sulphide
10	7.33	2,3-Hexandione	Buttery, cheesy, sweet, creamy	Ketone
11	8.83	Pyridine	Bitter, astringent, roasted, burnt	Heterocyclic N
12	11.83	Furfuryl methyl ether	Nutty, coffee grounds-like, rich, phenolic	Ether
13	12.53	2-Methylpyrazine	Nutty, roasted, chocolate	Pyrazine
14	12.70	Dihydro-2-methyl-3-furanone	Sweet, roasted	Ketone
15	14.96	2,5-Dimethylpyrazine	Nutty, roasted, grassy, corn	Pyrazine
16	15.27	2,6-Dimethylpyrazine	Nutty, sweet, fried	Pyrazine
17	15.59	2-Ethylpyrazine	Nutty, roasted	Pyrazine
18	16.03	2,3-dimethyl-Pyrazine	Nutty, roasted, green	Pyrazine
19	17.42	Dimethyl trisulphide	Onion	Sulphide
20	17.86	2-Ethyl-6-methylpyrazine	Roasted, hazelnut-like	Pyrazine
21	18.59	Trimethyl pyrazine	Nutty, roasted	Pyrazine
22	21.81	2-Furfural	Bread, almond, sweet	Aldehyde
23	22.94	Acetic acid	Sour	Organic acid
24	23.37	2-Acetylfuran	Balsamic-sweet	Furan
25	24.11	Pyrrole	Nutty, hay-like, herbaceous	Heterocyclic N
26	30.13	Furfuryl alcohol	Burnt	Alcohol
27	31.30	Butanoic acid	Sour	Organic acid
28	31.30	Hexanoic acid	Fatty-rancid, acrid-acid	Organic acid
29	35.11	2-Furfuryl methyl disulphide	Coffee-like	Sulphide
30	36.22	1-Furfurylpyrrole	Hay-like, mushroom-like, green	Heterocyclic N
31	40.76	Maltol	Caramel	Alcohol
32	41.16	1-(1-H-pyrrol-2-yl)ethanone	Nutty, musty	Ketone
33	41.81	Difurfuryl ether	Coffee-like, toasted odour	Ether
34	42.65	Phenol	Smoky	Phenolic
35	43.21	4-Ethyl-2-methoxyphenol	Smoky, spicy	Phenolic
36	45.25	Octanoic acid	Sweet cheesy	Organic acid
37	55.02	Indole	Burnt, mothball	Heterocyclic N

a Flament (2002).
