# Corrigendum to “Determination of volatile marker compounds of common coffee roast defects” [Food Chem. 211 (2016) 206–214]

**DOI:** 10.1016/j.foodchem.2020.126331

**Published:** 2020-06-15

**Authors:** Ni Yang, Chujiao Liu, Xingkun Liu, Tina Kreuzfeldt Degn, Morten Munchow, Ian Fisk

**Affiliations:** a Division of Food Sciences, University of Nottingham, Sutton Bonington Campus, Loughborough LE12 5RD, United Kingdom; b Department of Food Science, Faculty of Science, University of Copenhagen, Rolighedsvej 30, 1958 Frederiksberg C, Denmark; c The Specialty Coffee Association of Europe, Chelmsford, Essex, United Kingdom; d CoffeeMind Aps, Hansstedvej 35, 2500 Valby, Denmark

The authors regret, that the incorrect graph is showing as Figure 5a. The correct graph is shown below.

**Figure:**
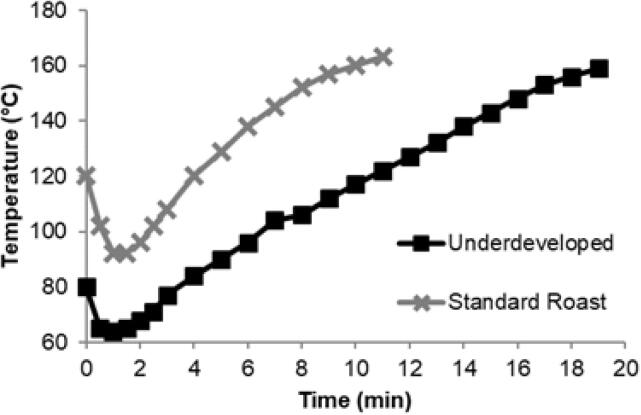

The authors would like to apologise for any inconvenience caused.

