# Supplementary material for: Determination of volatile marker compounds of common coffee roast defects
Source: Food Chem. 2016 Nov 15;211:206–14. doi: 10.1016/j.foodchem.2016.04.124 (PMC4914823; doi:10.1016/j.foodchem.2016.04.124)
Supplement: Supplementary data 1 [file mmc1.docx]

**Supplementary Tables and Figures**

Table S1 List of key roasting parameters used to generate coffee samples at six roasting profiles

| **Roasting Profile** | **Starting Temperature ^a^** | **Developing Time ^b^** | **Total Roasting Time** | **Agtron ^c^** |
| --- | --- | --- | --- | --- |
| Standard | 210 ◦C | 2min 40s | 11min 25s | 74.4 |
| Light | 210 ◦C | 10s | 8min 40s | 116.6 |
| Scorched | 275 ◦C | 1min 50s | 7min 40s | 66.0 |
| Dark | 220 ◦C | 4 min 45s | 13min 45s | 45.7 |
| Baked | 230 ◦C | 6min 20s | 18min | 68.3 |
| Underdeveloped | 135 ◦C | 2min 30s | 20min 20s | 74.9 |

^a^ air temperature measured when the beans entered the roaster;

^b^ time used from first crack (perceived as the first popping sound during roasting) to the end of roasting;

^c^ Agtron number is a spectrophotometric measure used to indicate the colour of the roasted beans.


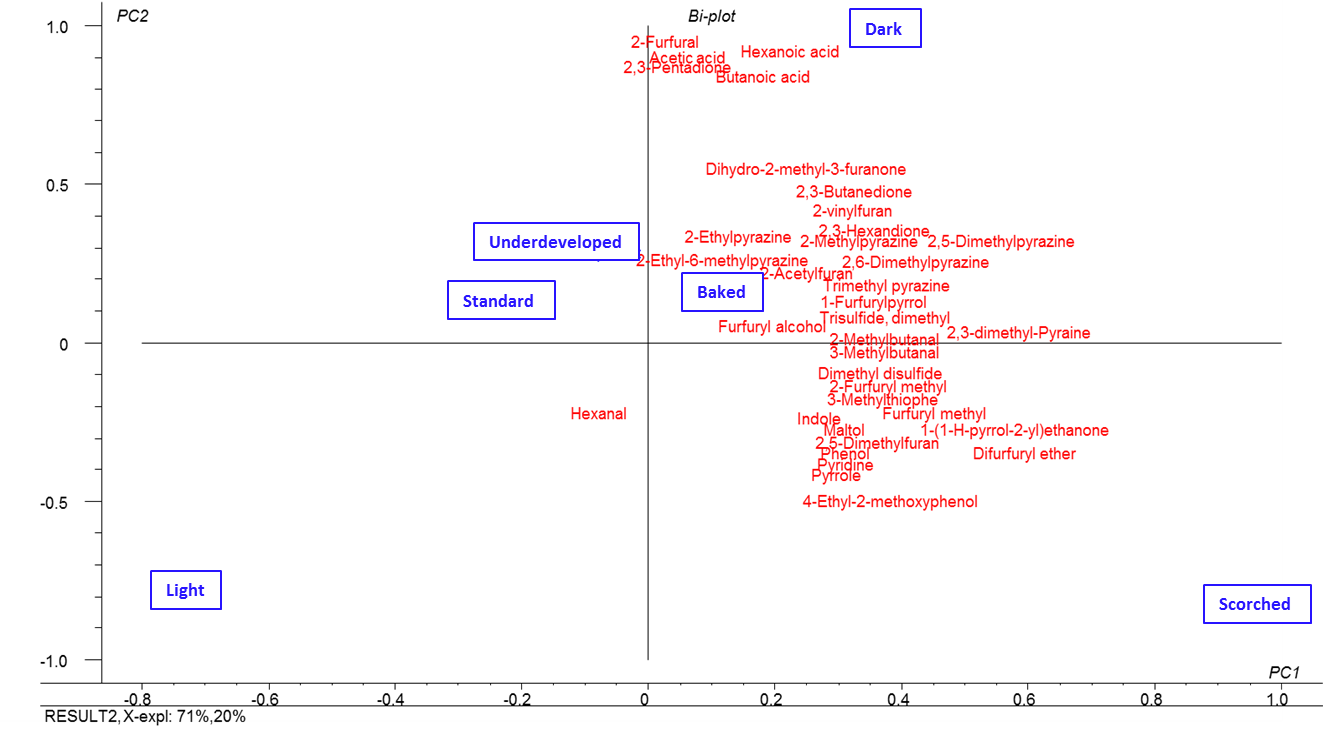


Figure S2: Principle component analysis bi-plot of the volatile aroma compliment of standard coffee and defect coffees analysed by GC-MS
